# Smoothed Particle Hydrodynamics Simulation of Wave Overtopping Characteristics for Different Coastal Structures

**DOI:** 10.1100/2012/163613

**Published:** 2012-07-31

**Authors:** Jaan Hui Pu, Songdong Shao

**Affiliations:** ^1^School of Engineering, Nazarbayev University, 53 Kabanbay Batyr Avenue, Astana 010000, Kazakhstan; ^2^School of Engineering, Design and Technology, University of Bradford, West Yorkshire BD7 1DP, UK

## Abstract

This research paper presents an incompressible smoothed particle hydrodynamics (ISPH) technique to investigate a regular wave overtopping on the coastal structure of different types. The SPH method is a mesh-free particle modeling approach that can efficiently treat the large deformation of free surface. The incompressible SPH approach employs a true hydrodynamic formulation to solve the fluid pressure that has less pressure fluctuations. The generation of flow turbulence during the wave breaking and overtopping is modeled by a subparticle scale (SPS) turbulence model. Here the ISPH model is used to investigate the wave overtopping over a coastal structure with and without the porous material. The computations disclosed the features of flow velocity, turbulence, and pressure distributions for different structure types and indicated that the existence of a layer of porous material can effectively reduce the wave impact pressure and overtopping rate. The proposed numerical model is expected to provide a promising practical tool to investigate the complicated wave-structure interactions.

## 1. Introduction

Many types of breakwaters have been developed for the purpose of shore and harbor protections. The common goal of such structures is to reduce the wave height and energy to an acceptable level in the coastal areas. When the structure is made of porous materials, additional wave energy is dissipated inside the structure due to the flow friction within the porous media. The wave overtopping of coastal structure has always been of great interest and many studies have been carried out to evaluate the flow overtopping discharge for different breakwater designs. For example, the European Overtopping Manual (EurOtop 2007) [1] provides a very comprehensive and practical tool for evaluating the wave overtopping for different sea defenses and has been widely used in the engineering field with sufficient accuracy. Besides, many other experimental and theoretical studies have also been performed to study the wave-breakwater interactions, including Brossard et al. [2], Muttray and Oumeraci [3], and Chen et al. [4].

Numerical modeling based on the Navier-Stokes (N-S) equations has the advantage of including the irregular seabed geometries, inhomogeneous porous media, nonlinear waves, and nonlinear friction forces. They are capable of calculating the flows inside the complex geometries to disclose very refined information about the velocity, pressure, turbulence, transport property, and so forth. The numerical models based on the 2D N-S type equations and the Reynolds averaged N-S (RANS) equations are possibly the most common to the study of wave-structure interactions and wave overtopping for engineering purposes, as the computational efforts are reasonably small, and the number of simplifying assumptions is considerably reduced as compared to other existing models. The relevant works include Qiu and Wang [5], Liu et al. [6], Huang and Dong [7], and Garcia et al. [8].

 In this paper, we propose an incompressible smoothed particle hydrodynamics (ISPH) model to study the wave interaction and overtopping for different breakwater designs. The SPH method was originally developed for the astrophysics by Monaghan [9] and recently commonly used to the fluid flows [10]. One of the great advantages of the SPH to model free surface flows is that the particles move in a Lagrangian coordinate, and the advection is directly calculated by the particle motion. Thus, free surfaces can be conveniently and accurately tracked by the particles without numerical diffusion, which is usually encountered in the traditional Eulerian approach. In the early simulations of fluid flows by the weakly compressible SPH [10], the incompressibility was realized through an equation of state so that the fluid was assumed to be slightly compressible. In this case, a large sound speed has to be introduced, which could easily cause problems of sound wave reflections at the solid boundaries, and the high sound speed could lead to the crippling CFL time-step constraint. In the ISPH conception [11, 12], the pressure is not a thermodynamic variable obtained from the equation of state, but obtained by way of solving a pressure Poisson equation derived from a semi-implicit algorithm. It has been demonstrated that both the computational efficiency and stability could be improved in the ISPH due to that a relatively larger time step can be used, and the particle fluctuation is reduced [13].

Here we use the ISPH model to study the wave overtopping of a coastal structure with different characteristics: vertical and sloping walls, with and without the protection of porous materials. The flow velocity field, turbulence, and pressure distributions will be compared for the different designs to evaluate their performance. Finally, the flow overtopping discharges will be validated against the available data published in the literature [6]. It is worth to mention that many of the state-of-the-art wave overtopping simulations have been carried out by researchers using either the mesh-based or mesh-free methods, such as in [14–18].

## 2. Principles of Incompressible SPH Model

### 2.1. Governing Equations

Following the work of Shao [11], the Lagrangian form of governing equations is used in the ISPH. In an SPH framework, the mass and momentum equations for the flow field are represented as follows:

(1) $$\begin{array}{c} \frac{1}{\rho }\frac{d\rho }{dt}+\nabla \cdot u=0, \end{array}$$

(2) $$\begin{array}{c} \frac{du}{dt}=-\frac{1}{\rho }\nabla P+g+\nu_{0}\nabla^{2}u+\frac{1}{\rho }\nabla \cdot \overset{\Rightarrow }{\tau }, \end{array}$$

where *ρ* = density, *t* = time, **u** = velocity, *P* = pressure, **g** = gravitational acceleration, *ν*_0_ = laminar viscosity, and $\overset{\Rightarrow }{\tau }$ = turbulence stress. It is noted that both (1) and (2) are written in the form of a full derivative on the left side of equations to enable an SPH formulation.

 The turbulence stress $\overset{\Rightarrow }{\tau }$ in (2) needs to be modeled to close the equation. In Liu et al. [6], the effect of turbulence is modeled by an improved *k* − *ε* model. Here a simple and effective eddy-viscosity-based subparticle scale (SPS) turbulence model originally developed by Gotoh et al. [19], which has been widely used in both the coastal and river hydrodynamics, is used to model the turbulence stress as:

(3) $$\begin{array}{c} \frac{\tau_{ij}}{\rho }=2\nu_{T}S_{ij}-\frac{2}{3}k\delta_{ij}, \end{array}$$

where *ν*_*T*_ = turbulence eddy viscosity, *S*_*ij*_ = strain rate of the mean flow, *k* = turbulence kinetic energy, and *δ*_*ij*_ = Kronecker's delta. We use the widely adopted Smagorinsky model [20] to calculate the turbulence eddy viscosity *ν*_*T*_ as follows:

(4) $$\begin{array}{c} \nu_{T}={\left(C_{s}\Delta X\right)}^{2}\left|S\right|, \end{array}$$

where *C*_*s*_ = Smagorinsky constant, which is taken as 0.1 in the paper, Δ*X* = particle spacing, which represents the characteristic length scale of the small eddies, and |*S*| = (2*S*_*ij*_ *S*_*ij*_)^1/2^ is the local strain rate.

To apply the above numerical model for the flows inside the porous materials, it is generally not easy to solve the N-S equations directly inside the pores. Thus, by following Gotoh and Sakai [21], the effect of a permeable layer is addressed by taking into account the additional external forces, namely, the drag forces, into the momentum equation (2). The drag forces due to the existence of the permeable layer can be written as follows:

(5) $$\begin{array}{c} F=-\frac{3C_{D}}{4\Delta X}\left|u\right|u, \end{array}$$

where *C*_*D*_ = drag coefficient due to the existence of porous materials. Shimizu and Tsujimoto [22] estimated the range of values of the drag coefficient to be between 1.0 and 1.5, based on the experiment of flow inside a permeable layer made by the vertical cylinders. In the current paper, a value of 1.25 was taken but we did not test the sensitivity of the value.

Although much more advanced porous flow treatment has been given in Shao [23], it was found that the above simple formulation can well address many kinds of the porous flows with enough accuracy. Besides, this approach was also successfully employed by Gotoh and Sakai [21] to study the plunging wave breaking on a permeable slope using the moving particle semi-implicit (MPS) modeling approach.

### 2.2. ISPH Solution Procedures

Following Shao [11], the ISPH model employs a two-step prediction/correction solution approach similar to the two-step projection method of Chorin [24] for solving the Navier-Stokes equations.

The prediction step is an explicit integration in time without enforcing the incompressibility. In this step, only the gravitational force, viscous/turbulence, and resistance forces in (2) and (5) are used and an intermediate particle velocity and position are obtained as:

(6) $$\begin{array}{c} \Delta u_{*}=\left(g+\nu_{0}\nabla^{2}u+\frac{1}{\rho }\nabla \cdot \overset{\Rightarrow }{\tau }-\frac{3C_{D}}{4\Delta X}\left|u\right|u\right)\Delta t,\\ u_{*}=u_{t}+\Delta u_{*},\\ r_{*}=r_{t}+u_{*}\Delta t, \end{array}$$

where Δ**u**_∗_ = increment of particle velocity during the prediction step, Δ*t* = time increment, **u**_*t*_ and **r**_*t*_ = particle velocity and position at time *t*, and **u**_∗_ and **r**_∗_ = intermediate particle velocity and position.

In the correction step, the pressure is used to update the particle velocity obtained from the prediction step

(7) $$\begin{array}{c} \Delta u_{**}=-\frac{1}{\rho_{*}}\nabla P_{t+1}\Delta t,\\ u_{t+1}=u_{*}+\Delta u_{**}, \end{array}$$

where Δ**u**_∗∗_ = increment of particle velocity during the correction step, *ρ*_∗_ = intermediate particle density calculated after the prediction step, and *P*_*t*+1_ and **u**_*t*+1_ = particle pressure and velocity at time *t* + 1.

Finally, the positions of particle are centered in time

(8) $$\begin{array}{c} r_{t+1}=r_{t}+\frac{\left(u_{t}+u_{t+1}\right)}{2}\Delta t, \end{array}$$

where **r**_*t*_ and **r**_*t*+1_ = positions of particle at time *t* and *t* + 1.

The pressure is implicitly calculated from the Poisson equation of pressure as follows:

(9) $$\begin{array}{c} \nabla \cdot \left(\frac{1}{\rho_{*}}\nabla P_{t+1}\right)=\frac{\rho_{0}-\rho_{*}}{\rho_{0}\Delta t^{2}}, \end{array}$$

where *ρ*_0_ = initial constant density at each of the particle in the beginning of computation. Equation (9) was derived from the combination of the mass and momentum equations (1) and (2), by enforcing the incompressibility of particle densities. It is analogous to that employed in the moving particle semi-implicit (MPS) method [25] in that the source term of the equation is the variation of particle densities, while it is usually the divergence of intermediate velocity fields in a finite difference method.

### 2.3. Basic SPH Theories and Formulations

The advantages of the SPH approach arise from its gridless nature. Since there is no mesh distortion, the SPH method can effectively treat the large deformations of free surface and multi-interface in a pure Lagrangian frame. In an SPH framework, the motion of each particle is calculated through the interactions with the neighboring particles using an analytical kernel function. All terms in the governing equations are represented by the particle interaction models, and thus the grid is not needed. For a detailed review of the SPH theories see Monaghan [9]. Among a variety of kernels documented in the literatures the spline-based kernel normalized in 2-D [9] is widely used in the hydrodynamic calculations. We use the following basic formulations for the proposed ISPH model.

The fluid density *ρ*_*a*_ of particle *a* is calculated by

(10) $$\begin{array}{c} \rho_{a}=\sum_{b}m_{b}W\left(\left|r_{a}-r_{b}\right|,h\right), \end{array}$$

where *a* and *b* = reference particle and all of its neighbors; *m*_*b*_ = particle mass, **r**_*a*_ and **r**_*b*_ = particle positions, *W* = interpolation kernel, and *h* = smoothing distance, which determines the range of particle interactions and is equal to 1.2 times of the initial particle spacing in the paper.

The pressure gradient assumes a symmetric form as:

(11) $$\begin{array}{c} {\left(\frac{1}{\rho }\nabla P\right)}_{a}=\sum_{b}^{}m_{b}\left(\frac{P_{a}}{\rho_{a}^{2}}+\frac{P_{b}}{\rho_{b}^{2}}\right)\nabla_{a}W_{ab}, \end{array}$$

where the summation is over all particles other than particle *a* and ∇_*a*_ *W*_*ab*_ = gradient of the kernel taken with respect to the positions of particle *a*. In a similar way, the velocity divergence of particle *a* is formulated by

(12) $$\begin{array}{c} \nabla \cdot u_{a}=\rho_{a}\sum_{b}m_{b}\left(\frac{u_{a}}{\rho_{a}^{2}}+\frac{u_{b}}{\rho_{b}^{2}}\right)\cdot \nabla_{a}W_{ab}. \end{array}$$

The turbulence stress in (2) is formulated by applying the above SPH definition of divergence as

(13) $$\begin{array}{c} {\left(\frac{1}{\rho }\nabla \cdot \overset{\Rightarrow }{\tau }\right)}_{a}=\sum_{b}m_{b}\left(\frac{{\overset{\Rightarrow }{\tau }}_{a}}{\rho_{a}^{2}}+\frac{{\overset{\Rightarrow }{\tau }}_{b}}{\rho_{b}^{2}}\right)\cdot \nabla_{a}W_{ab}. \end{array}$$

The Laplacian of pressure and laminar viscosity terms are formulated as a hybrid of a standard SPH first derivative with a finite difference approximation for the first derivative. They are also represented in the symmetrical forms as

(14) ∇·(1ρ∇P)a=∑bmb8(ρa+ρb)2 ×(Pa−Pb)(ra−rb)·∇aWab|ra−rb|2,

(15) (ν0∇2u)a=∑bmb2(νa+νb)ρa+ρb ×(ua−ub)(ra−rb)·∇aWab|ra−rb|2.

### 2.4. Treatment of Solid Boundary and Free Surface

In the ISPH computations, the free surface can be easily and accurately tracked by the fluid particles. Since there is no fluid particle existing in the outer region of the free surface, the particle density on the surface should drop significantly. A zero pressure is given to each of the surface particles.

The impermeable solid boundaries such as the horizontal sea bed and sloping sea walls are treated by the fixed wall particles, which balance the pressure of inner fluid particles and prevent them from penetrating the wall. The pressure Poisson equation is solved on these wall particles. The offshore boundary is the incident wave boundary, which is modeled by a numerical wave paddle composed of moving wall particles. In the computations, the frequency and amplitude of the numerical wave paddle are given so as to generate the desired incident waves. Most kinds of the practical waves can be easily generated by the SPH model. For a more detailed description of the boundary treatment in particle models, refer to [11, 21, 25].

## 3. Wave Overtopping for Different Breakwater Designs

### 3.1. Model Setup and Numerical Parameters

In this section, we use the developed ISPH model to study a practical engineering problem: the breaking wave overtopping on a caisson breakwater under different conditions, including with and without the protection by a porous armor layer and different slope geometries. We will investigate the overtopping mass rate, pressure, velocity, and turbulence features in front of the breakwater to study the flow characteristics. The computational setting is based on the laboratory experiment of Sakakiyama and Kayama [26] and the numerical computations of Liu et al. [6].

**Figure 1:**
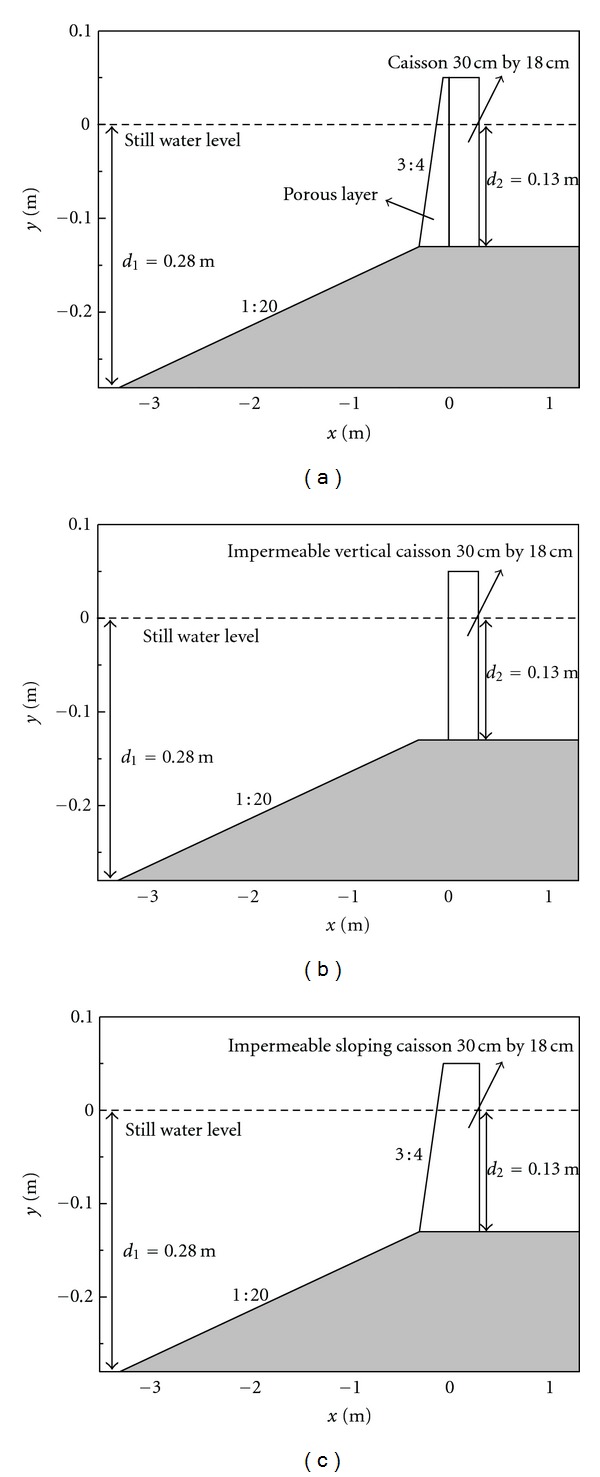
Sketch view of numerical setup for wave overtopping of different breakwaters: (a) with a permeable layer, (b) impermeable vertical wall, and (c) impermeable sloping wall.

The laboratory experiment used an impermeable caisson breakwater with a dimension of 30 cm × 18 cm and a layer of porous materials in front of the caisson. The effective porosity is 0.5, and the mean diameter of porous materials is 0.05 m. The ISPH model is used to reproduce the experiment of Sakakiyama and Kayama [26] in which the wave period was *T* = 1.4 s, wave height *H* = 0.105 m, and still water depth *d* = 0.28 m. A sketch view of the numerical setup including the caisson breakwater and a layer of porous armor units is shown in Figure 1(a), where the origin of coordinates is chosen at the intersection of the still water level and front wall of the caisson. The free surfaces were measured at several sections for more than 40 seconds in the experiment. The overtopped mass was also weighed to estimate the overtopping rate.

In the ISPH simulations, a smaller computational domain and shorter simulation time are used to reduce the computational effort. The computational domain is 5.3 m long and covers the caisson breakwater and a numerical wave paddle located at the offshore boundary. A uniform particle spacing of Δ*X* = 0.01 m is used and about 12,000 particles are involved in the computations. The spatial resolution in the ISPH run is similar to that used in the RANS computation of Liu et al. [6], who used a nonuniform grid of Δ*x*_min⁡_ = 0.01 m and Δ*y*_min⁡_ = 0.007 m. Because the leading reflected wave from the caisson reached the wave paddle about 7 seconds after the first wave was generated by the paddle, we impose the nonreflecting wave paddle of Hayashi et al. [27] to absorb the reflected waves to ensure that the quasisteady condition can be attained. The total simulation time is *t* = 13 seconds in the incompressible SPH computations.

To further demonstrate the protective role of the porous armor layer, two alternative numerical experiments are also performed to compare the flow velocity, turbulence, and pressure characteristics in front of the caisson breakwater without the protection of the porous layer. The design of the problem follows Liu et al. [6]. In the first case, the porous layer is completely removed so the waves impact directly on the vertical caisson wall. In the second case, the porous layer is replaced by an impermeable material, so the impermeable material and the caisson become a single structure like an impermeable sloping seawall. The numerical settings for the additional two cases are shown in Figures 1(b) and 1(c), respectively.

Here we should emphasize that in the work of Shao [23], the detailed breaking wave running up and overtopping characteristics for the porous case have been discussed and the wave profiles have been validated against the benchmark data. In the current paper, the focus will be the comparisons of flow characteristics for the different design scenarios and especially the flow overtopping rate, which is a key parameter in the practical breakwater design.

### 3.2. Discussions of Flow Velocity Field for Different Cases

**Figure 2:**
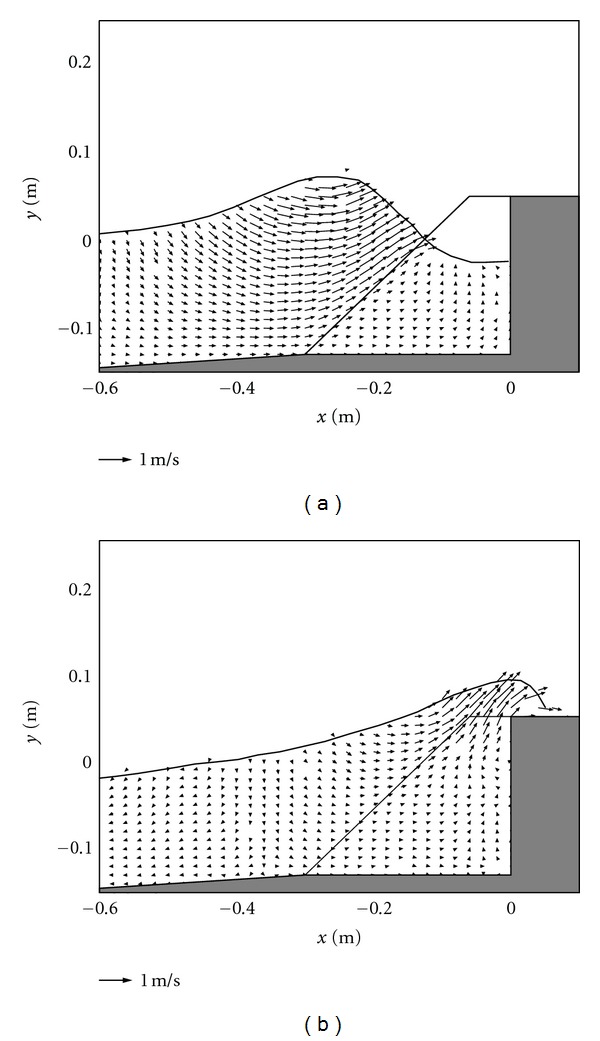
Velocity fields during (a) wave interaction and (b) overtopping on caisson breakwater protected by a porous layer.

**Figure 3:**
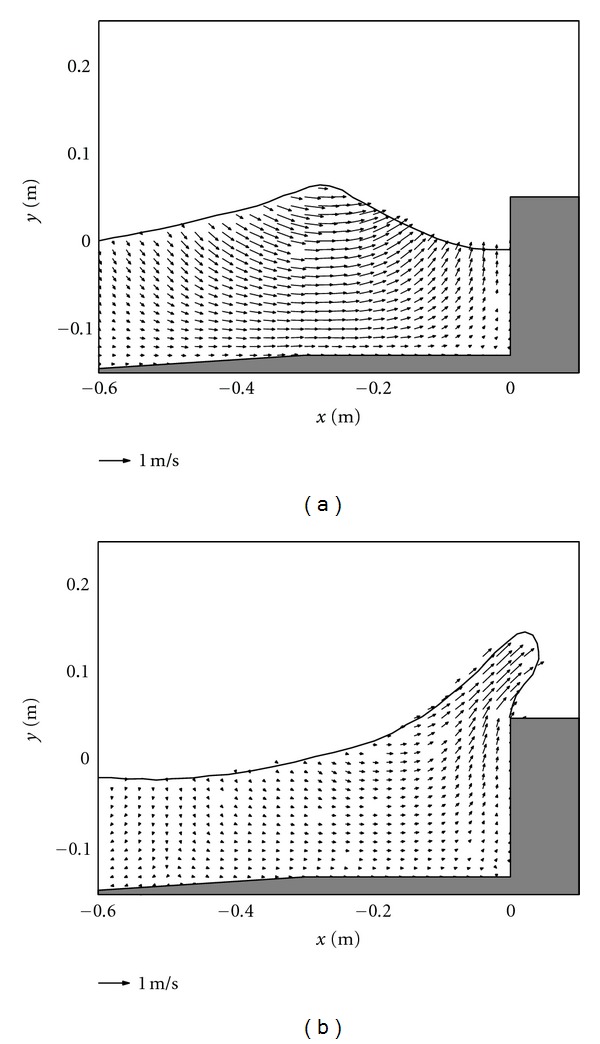
Velocity fields during (a) wave interaction and (b) overtopping on impermeable vertical caisson breakwater.

**Figure 4:**
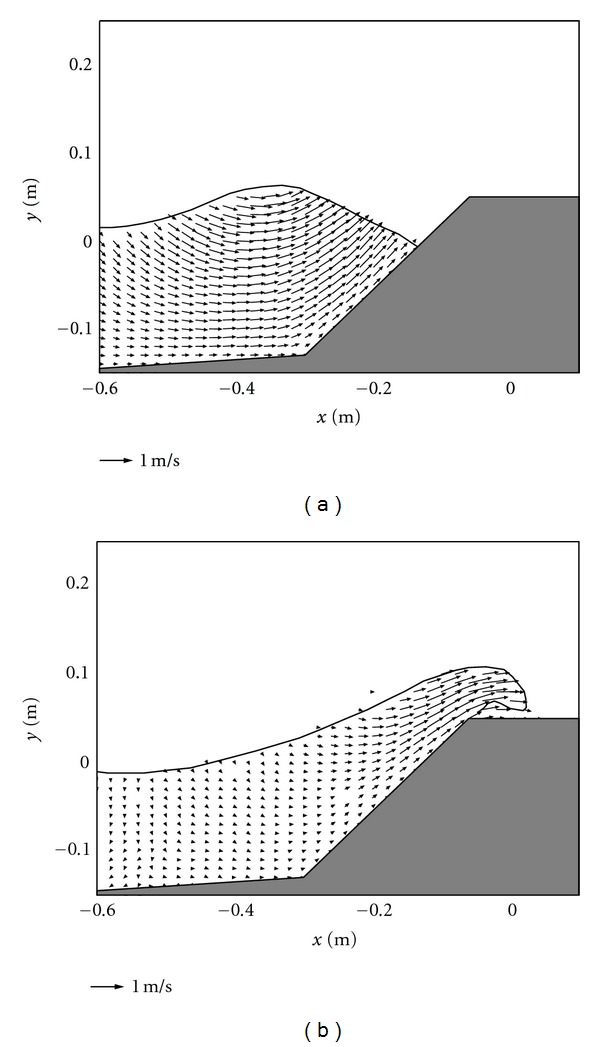
Velocity fields during (a) wave interaction and (b) overtopping on impermeable sloping caisson breakwater.

According to the ISPH simulations, the flow velocity fields during the wave interaction and overtopping on the caisson breakwater are shown in Figures 2, 3, and 4, respectively, corresponding to the case of the porous layer, impermeable vertical, and sloping walls. From Figures 2(a) and 2(b), the velocity fields demonstrate the protective role of the porous armor layer in absorbing the wave energy and attenuating the flow impact on the caisson, where it acts as an equivalent buffer layer. From the figure, it is shown that the velocities decrease towards the core of the armor units and become nearly zero near the toe of the caisson. Thus, the scour of the caisson by the continuous wave actions can be greatly reduced due to the use of the armor unit. This phenomenon has also been well described by Liu et al. [6] in their numerical simulations by using an advanced RANS approach [28].

For Figures 3(a) and 3(b) in case of the impermeable vertical wall, due to the absence of the porous armor units, it lacks an efficient mean to absorb the wave energy. Compared with the case by using the porous armor layer in Figure 2, the flow motion in Figure 3 is much stronger. When the wave overtopping starts to occur, both the vertical velocities and vertical accelerations are quite large in front of the caisson. Therefore, the potential scouring at the foot of the caisson becomes severe in this case. In addition, the size of the overtopping jet is also much larger and thus carries a lot of energy, which could threaten the safety of the onshore areas.

For Figures 4(a) and 4(b) in case of the impermeable sloping wall, although this kind of design has a similar physical geometry as that used in Figure 2, the impermeable sloping wall prevents the wave energy dissipation and provides a chance for the wave to run up. Although the size and intensity of the overtopping jet are relatively smaller as compared with the vertical jet generated in Figure 3 in a vertical caisson without any protection, the wave overtopping capacity is actually enhanced in Figure 4, due to that the flow can maintain sufficiently large horizontal momentum for the subsequent overtopping process.

### 3.3. Discussions of Flow Pressure Field for Different Cases

**Figure 5:**
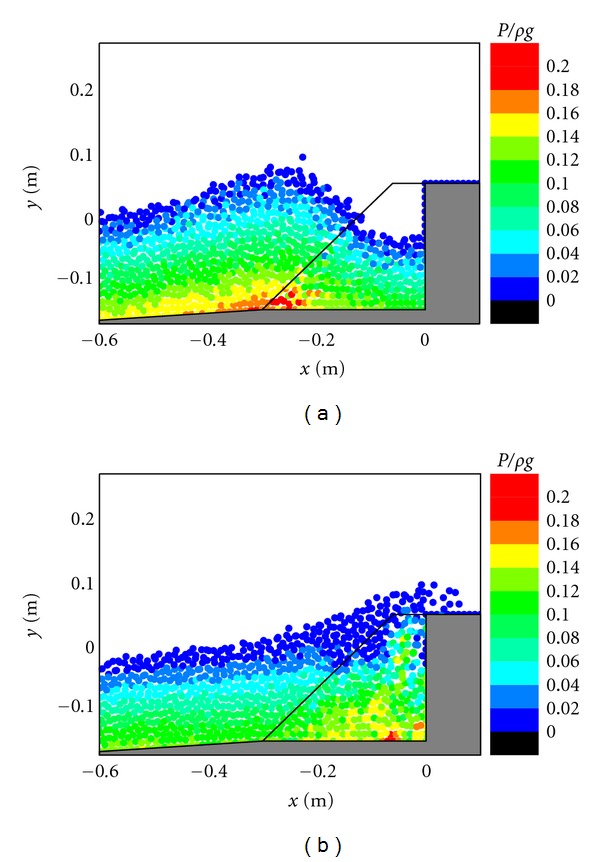
Pressure fields during (a) wave interaction and (b) overtopping on caisson breakwater protected by a porous layer.

**Figure 6:**
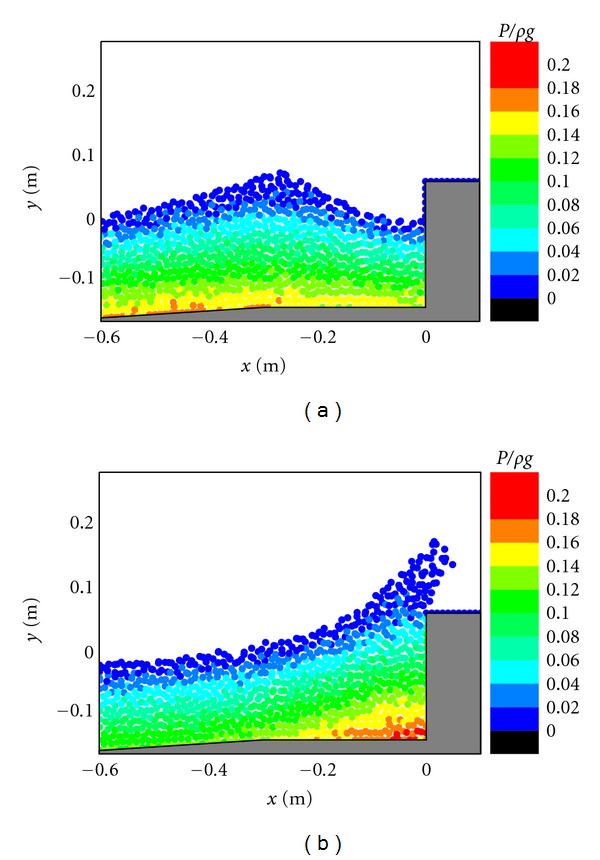
Pressure fields during (a) wave interaction and (b) overtopping on impermeable vertical caisson breakwater.

**Figure 7:**
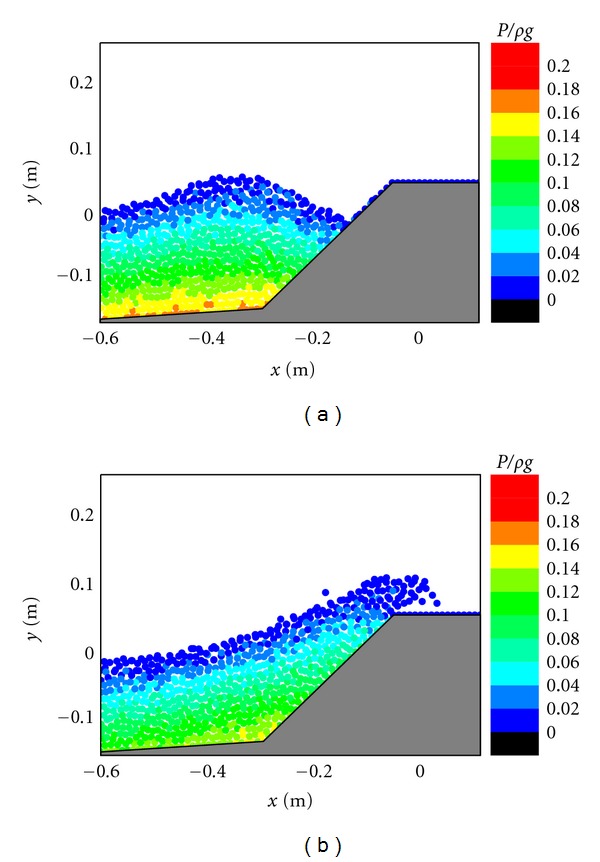
Pressure fields during (a) wave interaction and (b) overtopping on impermeable sloping caisson breakwater.

The impact pressure on the caisson breakwater is another very important topic in the breakwater stability and scouring problems. For studying this, the pressure fields in front of the caisson breakwater are shown in Figures 5, 6, and 7, respectively, corresponding to the three different cases. In the figures, the pressure values have been normalized by *ρg* to represent the normalized pressure head. For Figure 5, in case of the existence of the porous layer, it is shown that the pressure generally follows a hydrostatic distribution before the wave impacts on the structure as shown in Figure 5(a). However, as the wave front approaches the caisson and overtops on the crest, the fluid particles experience a vertical acceleration that results in a slightly larger pressure than the hydrostatic value. As shown in Figure 5(b), the maximum pressure seems to be resulted from the wave impact because the pressure distributions in front of the caisson do not exactly follow the hydrostatic law. Besides, the range of impact pressures is greatly reduced due to the protection of the porous armor layer. The computed pressure patterns in Figure 5 generally agree with the RANS results of Liu et al. [6].

The pressure fields presented in Figure 6 indicate that stable pressure calculations are also achieved for the simplification of the physical problem without considering the porous flow. Although no much difference in the pressure patterns is observed during the wave approaching the caisson breakwater as shown in Figures 6(a) and 5(a), the pressure patterns seem quite different during the wave overtopping on the caisson as shown in Figures 6(b) and 5(b). Without the protection by the porous materials, the pressures increase more rapidly and the high pressure regions are more widely spread at the foot of the caisson, which can pose a great threat to the structure stability in practice. For Figure 7, in case of the impermeable sloping wall, although the maximum pressure is smaller than that in the porous case in Figure 5, the wave overtopping capacity can significantly increase due to the reasons as mentioned before.

 The above stable pressure simulations demonstrated the robustness of the ISPH model presented in the paper. It is well known that the pressure fluctuation is a common problem in most particle modeling approaches, which arises from the particle interactions and inevitably leads to the particle fluctuations. Such a problem has been reported in the widely used weakly compressible SPH approach [9], and additional numerical treatments are needed to address this problem. In the ISPH computation, we can directly obtain very smoothed and reasonable pressure fields without any numerical smoothing. This is due to the fact that, in the ISPH formulation, the pressure is calculated through a strict hydrodynamic formulation. So the incompressible approach could represent a promising particle modeling technique for different hydrodynamic problems.

### 3.4. Discussions of Flow Turbulence Field for Different Cases

**Figure 8:**
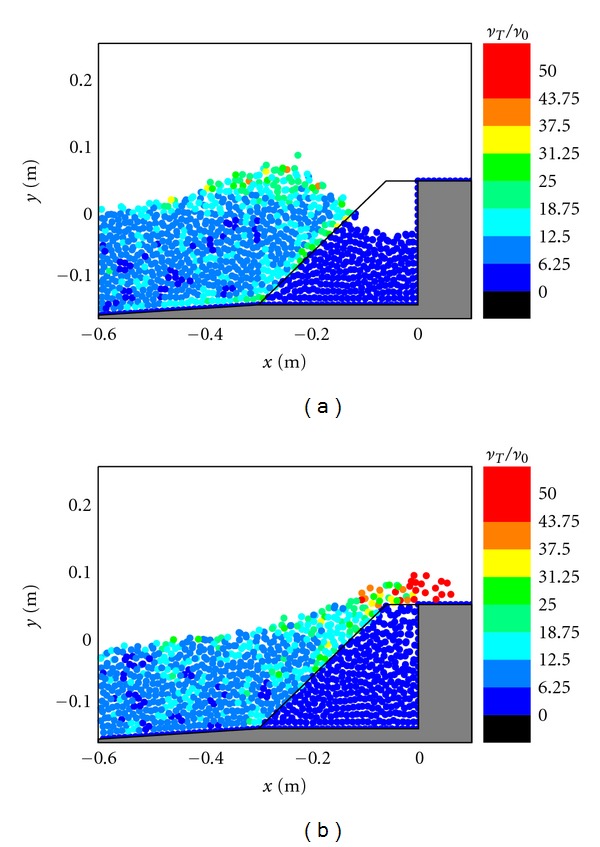
Turbulence fields during (a) wave interaction and (b) overtopping on caisson breakwater protected by a porous layer.

**Figure 9:**
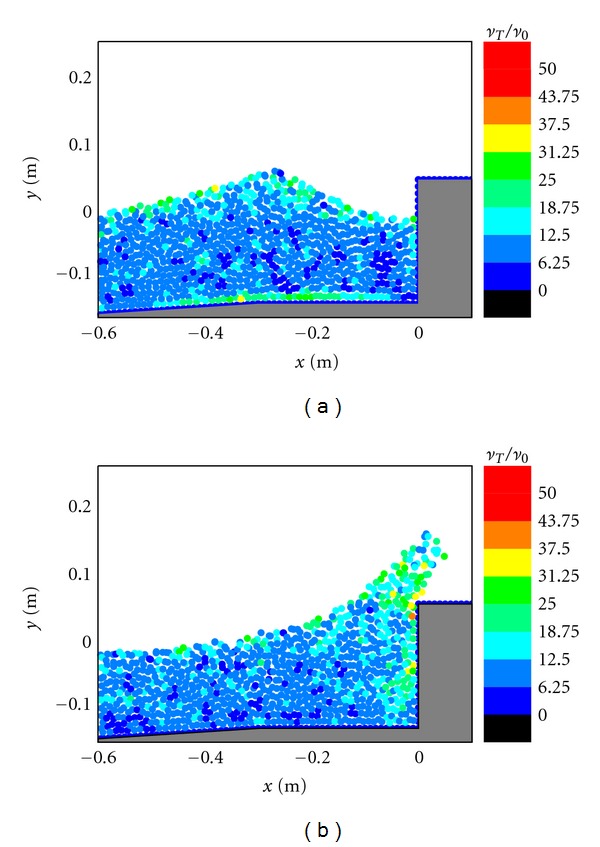
Turbulence fields during (a) wave interaction and (b) overtopping on impermeable vertical caisson breakwater.

**Figure 10:**
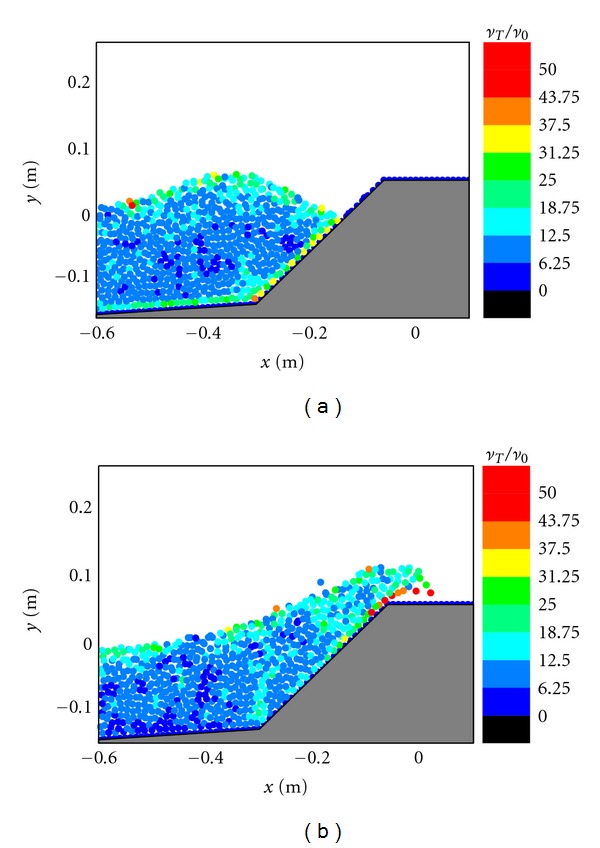
Turbulence fields during (a) wave interaction and (b) overtopping on impermeable sloping caisson breakwater.

The flow turbulence fields in front of the caisson are shown in Figures 8, 9, and 10, respectively, for the three different design cases. In the figures, the turbulence eddy viscosity values have been normalized by the laminar viscosity to represent the equivalent turbulence intensity. As shown in Figure 8, the turbulence levels inside the porous layer are very low and close to zero, because the porous material dampens most of the flow energy. Figure 8 also indicates that the high turbulence areas almost concentrate on the wave crest and the overtopping wave front, where the flow velocity is also the largest. In Figure 8(b), it is quite obvious that the highest turbulence areas concentrate on the overtopping jet. Since the turbulence is related to the energy dissipation, we can reasonably conclude that most of the kinetic energy of the overtopping wave is dissipated by the turbulence generation, and thus the overtopping intensity can be greatly reduced (We will have the detailed comparison of the overtopping mass rate in the following section to support this).

The computed flow turbulence fields in Figures 9 and 10 for the two impermeable cases have demonstrated a similar evolution pattern as that in the case with the protection of a porous armor layer in Figure 8, that is, the high turbulence regions almost concentrate on the wave crest and overtopping jet, as well as the lower solid boundary. The overall turbulence intensity in the overtopping front for the impermeable sloping wall in Figure 10(b) is higher than that in the impermeable vertical wall case in Figure 9(b).

### 3.5. Comparisons of Wave-Overtopping Load for Different Cases

**Figure 11:**
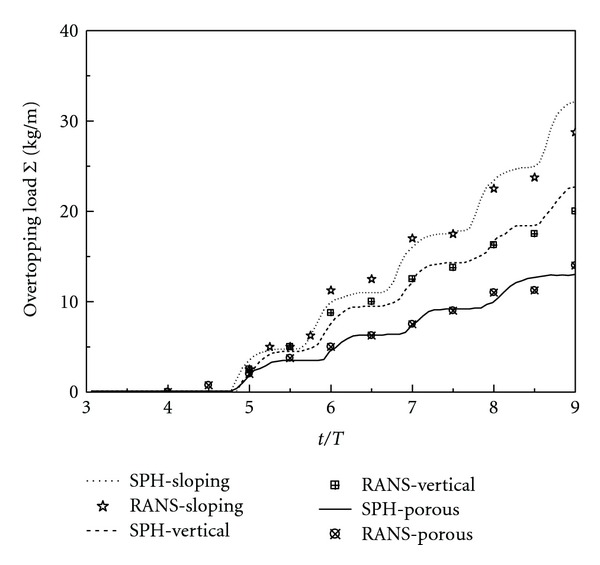
Time history of wave overtopping load for different breakwater designs.

The wave overtopping load is an important parameter to evaluate the performance of the breakwater design in practice. In order to quantitatively analyze the effectiveness of three different designs of the caisson breakwater, the time history of the wave-overtopping load for each design is shown in Figure 11, based on the ISPH computations. As a comparison, the numerical results computed from the RANS model of Liu et al. [6] are also shown. Regardless of some differences in the detailed velocity, turbulence, and pressure fields computed by the two numerical models, the overall agreement in the wave-overtopping mass is quite excellent. The relatively large deviation is found for the computations with the presence of the porous armor layer, which could be attributed to the different treatments of the turbulence boundary conditions and the drag forces by the two models.

Figure 11 indicates that the caisson breakwater protected by the porous armor layer (denoted as “SPH-Porous” in the figure) has the smallest wave overtopping. By using the statistical analysis, it is calculated that the caisson without the armor units (denoted as “SPH-Vertical”) increases the wave-overtopping load by about 55% and the use of an impermeable sloping wall (denoted as “SPH-Sloping”) increases the overtopping load by about 105%. This is quite close to the documented values of 45% and 100%, respectively, computed by Liu et al. [6]. The figure has provided a quantitative measurement to show the effectiveness of the porous materials in attenuating the incident wave energy and protecting the coastal structure from severe wave attack.

## 4. Conclusions

An incompressible smoothed particle hydrodynamics model has been used to evaluate the wave-breakwater interactions and wave overtopping. The SPH numerical scheme has the advantage of treating the free surfaces and complex solid boundaries in an easy and accurate way. All of the computations were made by using a CPU 2.13 G and RAM 1.0 G laptop. A single run was finished within 6–8 hours by employing 12,000 particles for a wave simulation of 13 seconds (with an averaged time step of 0.001 s).

The numerical model was applied to the problem of a breaking wave interacting and overtopping on a caisson breakwater. The computed wave overtopping mass rate is in good agreement with the available numerical results computed from an RANS model. The numerical results of the flow velocities, pressures, and turbulence quantities demonstrated that the armor units play an important role in dissipating the wave energy and stabilizing the caisson breakwater. According to the numerical study of different designs, it is shown that the overtopping mass can be reduced by about 55% and 105%, respectively, as compared with a similar design of the caisson breakwater without any protection, or with an attached impermeable sloping seawall.

## Notations

*C*_*D*_: Drag coefficient
*C*_*s*_: Smagorinsky constant
*d*: Still water depth
**F**: Drag force caused by porous media
**g**: Gravitational acceleration
*h*: SPH kernel smoothing distance
*H*: Wave height
*k*: Turbulence energy
*m*: Particle mass
*P*: Pressure
**r**: Position vector
|*S*|: Local strain rate
*S*_*ij*_: Strain rate of mean flow
*T*: Wave period
**u**: Velocity vector
*W*: SPH interpolation kernel
*δ*_*ij*_: Kronecker's delta
Δ*x*_min⁡_: Horizontal grid spacing in RANS
Δ*X*: Particle spacing
Δ*t*: Time increment
Δ**u**: Change in velocity
Δ*y*_min⁡_: Vertical grid spacing in RANS
*ν*_0_: Laminar viscosity
*ν*_*T*_: Turbulence eddy viscosity
*ρ*: Fluid density
*ρ*_0_: Initial constant density
$\overset{\Rightarrow }{\tau }$: Turbulence stress.

## Subscripts and Symbols

*a*: Reference particle
*b*: Neighboring particle
*t*: Time
∗: Intermediate value
∗∗: Corrected value.
